# Computational approaches for prediction of pathogen-host protein-protein interactions

**DOI:** 10.3389/fmicb.2015.00094

**Published:** 2015-02-24

**Authors:** Esmaeil Nourani, Farshad Khunjush, Saliha Durmuş

**Affiliations:** ^1^Department of Computer Science and Engineering, School of Electrical and Computer Engineering, Shiraz UniversityShiraz, Iran; ^2^School of Computer Science, Institute for Research in Fundamental Sciences (IPM)Tehran, Iran; ^3^Computational Systems Biology Group, Department of Bioengineering, Gebze Technical UniversityKocaeli, Turkey

**Keywords:** protein-protein interaction, pathogen-host interaction (PHI), computational PHI prediction, machine learning, data mining

## Abstract

Infectious diseases are still among the major and prevalent health problems, mostly because of the drug resistance of novel variants of pathogens. Molecular interactions between pathogens and their hosts are the key parts of the infection mechanisms. Novel antimicrobial therapeutics to fight drug resistance is only possible in case of a thorough understanding of pathogen-host interaction (PHI) systems. Existing databases, which contain experimentally verified PHI data, suffer from scarcity of reported interactions due to the technically challenging and time consuming process of experiments. These have motivated many researchers to address the problem by proposing computational approaches for analysis and prediction of PHIs. The computational methods primarily utilize sequence information, protein structure and known interactions. Classic machine learning techniques are used when there are sufficient known interactions to be used as training data. On the opposite case, transfer and multitask learning methods are preferred. Here, we present an overview of these computational approaches for predicting PHI systems, discussing their weakness and abilities, with future directions.

## Introduction

Many studies concerning identification of protein interactions and their associated networks were published (Aloy and Russell, 2003). Most of the previous studies were primarily focused on determining protein-protein interactions (PPIs) within a single organism (intra-species PPI prediction), while the prediction of PPIs between different organisms (inter-species PPI prediction) has recently emerged. Inter-species interactions may take many forms; in this survey, however, we focus on PPIs between pathogens and their hosts. Pathogen-host interaction (PHI) prediction is worthwhile to enlighten the infection mechanisms in the scarcity of experimentally-verified PHI data. Interactions between pathogen and host proteins allow pathogenic microorganisms to manipulate host mechanisms in order to use host capabilities and to escape from host immune responses (Dyer et al., 2010). Therefore, a complete understanding of infection mechanisms through PHIs is crucial for the development of new and more effective therapeutics.

Despite the critical need to improve the PHI knowledge, current progress is not adequate, suffering from scarcity of available experimental PHI data. Reliable experimental methods are time-consuming and expensive, making it unjustifiable to evaluate all possible PHIs. For instance, considering about 26,000 human proteins paired with a few thousands of pathogen proteins lead to millions of protein pairs to test experimentally. Scarce verified interactions are collected within a number of databases like HPIDB (Kumar and Nanduri, 2010), PATRIC (Wattam et al., 2014), PHISTO (Durmuş Tekir et al., 2013), VirHostNet (Navratil et al., 2009), and VirusMentha (Calderone et al., 2014). At this point, computational approaches come to help by predicting putative PHIs. In this paper, we concentrate on these computational studies, which are mandatory for enriching the available data and consequently increasing the pace of research in the field. The methods which were successfully applied specifically for PHI prediction in the literature are categorized based on pathogen-host systems in Table 1.

**Table 1:** **Computational studies for prediction of PHIs**.

**Pathogen-host system**	**References**
*Plasmodium falciparum*-Human	Krishnadev and Srinivasan, 2008
	Lee et al., 2008
	Wuchty, 2011
	Dyer et al., 2007
*Helicobacter pylori*-Human	Kim et al., 2007; Tyagi et al., 2009
Hepatitis C virus (HCV)-Human	Cui et al., 2012; Zheng et al., 2014
Phage T4-*Escherichia coli*	Krishnadev and Srinivasan, 2011
Phage lambda-*E. coli*	Krishnadev and Srinivasan, 2011
*C. albicans*-Zebrafish	Wang et al., 2013
*E. coli*-Human	Krishnadev and Srinivasan, 2011
*Plasmodium berghei*-Mouse	Reid and Berriman, 2013
*Plasmodium berghei*-Insect vector (Mosquito)	Reid and Berriman, 2013
Oral microbial-Human	Coelho et al., 2014
*Salmonella*-Human	Krishnadev and Srinivasan, 2011
	Arnold et al., 2012
	Kshirsagar et al., 2012
	Kshirsagar et al., 2013b
	Schleker et al., 2012a
	Mei and Zhu, 2014
	Schleker et al., 2014 (Review)
*Mycobacterium Tuberculosis* H37Rv-Human	Zhou et al., 2014
*Yersinia pestis*-Human	Krishnadev and Srinivasan, 2011
	Kshirsagar et al., 2012
	Kshirsagar et al., 2013b
*Mycobacterium apicomplexa* and *Mycobacterium kinetoplastida*-Human	Davis et al., 2007
*Xanthomonas oryzae*-Rice	Kim et al., 2008
*HTLV*-Human	Mei, 2014
HIV1-Human	Evans et al., 2009
	Tastan et al., 2009
	Mei, 2013
	Qi et al., 2010
	Dyer et al., 2011
	Ray et al., 2012
	Doolittle and Gomez, 2010
	Nouretdinov et al., 2012
	Mukhopadhyay et al., 2010, 2012, 2014
	Mondal et al., 2012
36 viral species-Human	Franzosa and Xia, 2011
Influenza A NS1–Human	De Chassey et al., 2013
HPV16–Human	Dong et al., in press
*Bacillus anthracis*-Human	Kshirsagar et al., 2013b
*Francisella tularensis*-Human	Kshirsagar et al., 2013b
Dengue virus-Human	Doolittle and Gomez, 2011
	Segura-Cabrera et al., 2013
Insect vector *A. aegypti*-Human	Doolittle and Gomez, 2011
*Salmonella*-Arabidopsis	Schleker et al., 2012a
	Schleker et al., 2014 (Review)
Human papilloma viruses (HPV)-Human	Cui et al., 2012
*R. solanacearum*-Arabidopsis	Li et al., 2012
*Y. pestis, M. tuberculosis, C. diphtheriae, C. ulcerans, E. coli, and C. pseudotuberculosis*-Human, Goat, Sheep, and Horse	Barh et al., 2013

Considering the relative availability of interaction data for HIV-Human system, notable number of studies are dedicated to this pathogen. Some other viral and bacterial pathogens are investigated and human is the main target as the host for investigation. Computational methods for predicting PHIs exploit known protein and domain interactions, and information on sequence of proteins. Network topology measures can complement these data. For instance, targeting hubs and bottleneck proteins in human PPI network by pathogen proteins is a well-accepted idea (Dyer et al., 2008; Durmuş Tekir et al., 2012; Schleker and Trilling, 2013; Zheng et al., 2014), though, they are not the sole targeted proteins (Chen et al., 2012). Classic machine learning methods are valuable remedy for cases where enough data for training are available. However, valuable efforts have recently been performed to apply these techniques for situations suffer from scarcity of known interaction data using machine learning based methods as transfer and multitask learning (Xu et al., 2010; Kshirsagar et al., 2013a,b).

In PPI prediction studies, methods specific for intra-species interactions are usually used. On the other hand, concentrating on the interactions between different organisms is a young branch of this field. The traditional methods cannot be applied here, their adaptation or devising new approaches would be mandatory.

## Machine learning and data mining based approaches

Applying machine learning techniques to bioinformatics is a well-accepted idea (Baldi and Brunak, 2001), which includes early efforts for PPI predictions (Bock and Gough, 2001). These methods utilize available PPI data as features for training and classifying interacting and non-interacting protein pairs. Both semi-supervised and supervised learning are used for PHI prediction. A Supervised method, which exploits exclusively labeled data, is applied in Tastan et al. (2009) integrating 35 features within eight groups using Random Forest (RF) classifier to deal with noisy and redundant features. The semi-supervised extension of their work is presented in Qi et al. (2010) which discarded 17 attributes from the feature vector that is related to determining 17 HIV-1 proteins. However, they have gained better performance through incorporating likely interactions (called “partially labeled”), which do not have sufficient evidence to be categorized as direct interaction. The same classifier is used as a quality control in Wuchty (2011), where a RF classifier assesses the quality of candidate interactions, obtained by discovering homologous and conserved interactions. The author filters the predicted results based on expression and molecular properties.

Conformal prediction is used in Nouretdinov et al. (2012) and the results are compared with those of Tastan et al. (2009) to assess the predictions. This method evaluates the conformance of new pairs with interacting pairs using a method called non-conformity measure (NCM) which shows distinction measure of an example regarding others. Their approach also allows the user to determine confidence level for prediction.

**Figure 1:**
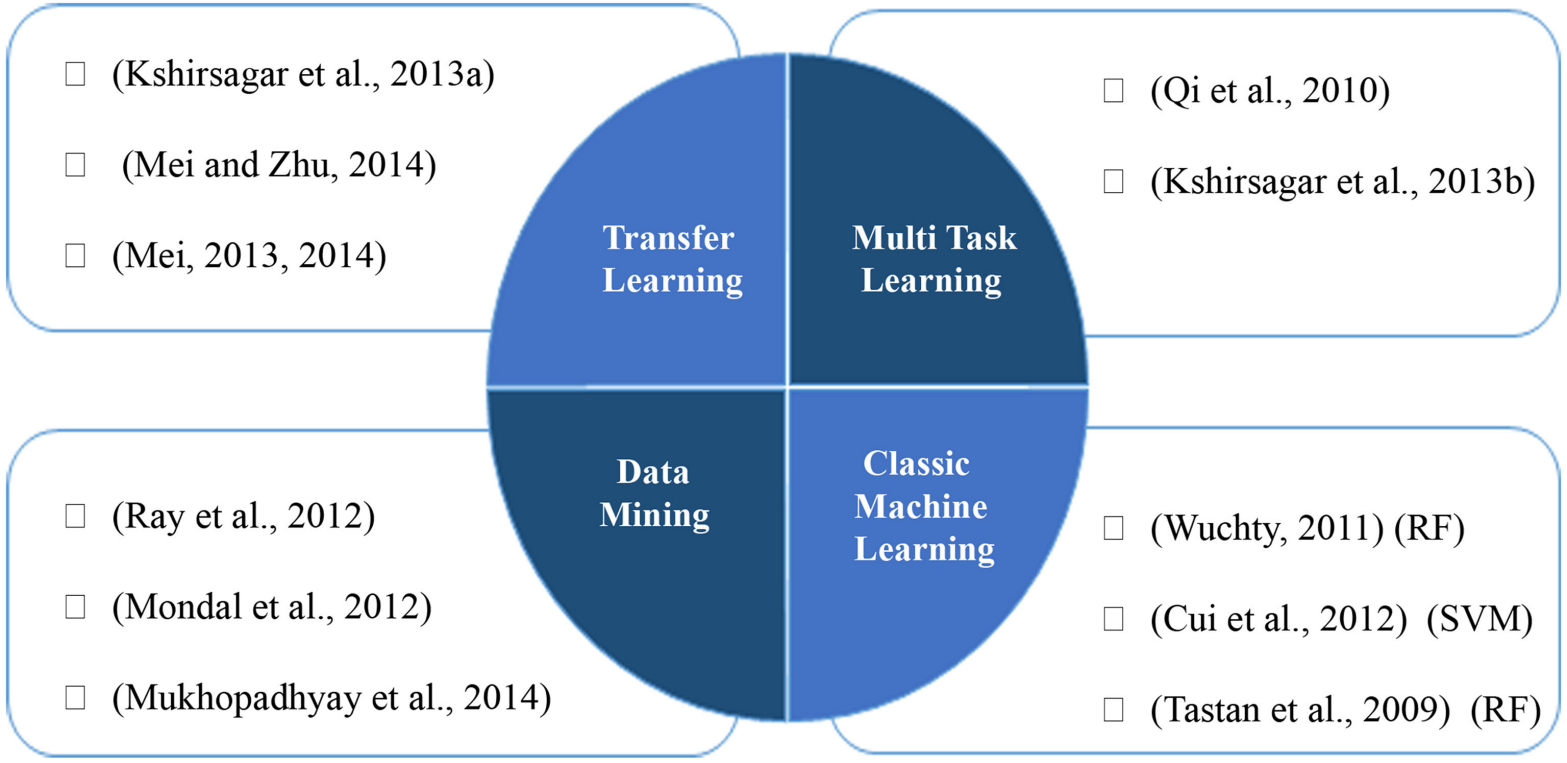
**Machine learning and data mining based approaches for prediction of PHIs**.

SVM based approaches as a famous classifier are successfully applied in PHI prediction studies (Kshirsagar et al., 2013a; Mei, 2013). Cui et al. (2012) presents a SVM based approach, which uses a fixed length feature vector, indicating relative frequency of consecutive amino acids in the protein sequence. We categorize the machine learning and data mining based approaches in Figure 1.

### Transfer and multitask learning approaches

One of the promising remedies to tackle the problem of data scarcity is eliciting and transferring data from related domains to desired formulation. Multitask learning uses commonalities among different domains and learn problem simultaneously between them within a shared task formulation, which leads to better performance rather conducting learning task on individual domain. A review paper, Xu and Yang (2011) presents some of the studies utilizing this idea in bioinformatics. For PPI prediction, a method was proposed in Xu et al. (2010) which uses collective matrix factorization originally proposed by Singh and Gordon (2008) to transfer knowledge from a relatively dense PPI network called “source” for predicting new PPIs in a sparse target PPI network. Their goal is to predict intra-species pathogen PPIs as target with the aid of human PPIs as source network through defining a similarity matrix to act as a bridge between them. Another study conducts three different individual classifiers on three GO features (molecular functions, cellular localization, and biological processes) on available protein features and at the same time three classifiers on alternative homolog features to exploit transfer learning. An ensemble classifier produces final result using weighting probability outputs of individual classifiers (Mei, 2013). They applied relatively same idea using a multi instance AdaBoost method to transfer homolog feature as the second instance of proteins (Mei, 2014; Mei and Zhu, 2014). A combination of supervised and semi-supervised approaches is proposed by Qi et al. (2010) through multitask learning. Semi-supervised task on partially positive labels is conducted to improve the supervised classification which trains multi-layer perceptron using labeled data. Another multitask formulation is used in Kshirsagar et al. (2013b) to integrate knowledge from different pathogen-host systems to increase the prediction power of the combined model. Each task is formulated as predicting PHI data between each pathogen and its host. To define similarity between tasks and transfer shared knowledge, they assume that similar pathogens tend to target same biological process in human. In other words, “commonality hypothesis” is introduced that assumes pathway membership of human proteins in positive PHIs should be similar between different tasks. To implement this idea, optimization problem is conducted and dissimilarities are penalized in the objective function. They use transfer learning in Kshirsagar et al. (2013a) for the cases where no known interaction is available by exploiting precisely chosen instances from a source task.

### Data mining based approaches

Machine learning based methods which formulate PPI prediction as a classification task use both interacting and non-interacting protein pairs as positive and negative classes, respectively. Constructing negative class is not straightforward due to the fact that there is no experimentally verified non-interacting pair. This has motivated some studies to overcome this problem by removing the need for negative data through using alternative methods (Mukhopadhyay et al., 2010, 2012, 2014; Mondal et al., 2012; Ray et al., 2012). They integrate bi-clustering with association rule mining, utilizing only positive samples to predict virus-human interactions.

### Utilized features

Various studies utilize different sets of biological information through data integration to improve the prediction performance. However, it should be noted that making use of a lot of features without enriching training data may lead to over fitting in the model (Mei, 2014). Table 2 summarizes the utilized features within different studies on PHI prediction, providing all the cataloged feature information is not always possible for all pathogen systems. Furthermore, various features claimed to have different predictive effects in PHI prediction. Outperforming other features was the motivation for some studies to use GO features in PHI prediction (Mei, 2013, 2014) while features extracted from protein sequences, reported as not promising (Yu et al., 2010).

**Table 2:** **Summary of the exploited features for prediction of PHIs**.

**Utilized feature**	**Description**	**References**
Domain and motif information	Set to be 1 every domain pair of each PPI in a binary feature vector of all possible domain pairs	Dyer et al., 2011
	Count possible interacting domains between pathogen and host proteins using domain interactions database (3DID)	Kshirsagar et al., 2012, 2013b
	Functional sequence motifs from ELM database checked in HIV-1 sequence	Tastan et al., 2009; Qi et al., 2010; Nouretdinov et al., 2012
	Suppose protein pairs as interacting when they have one or more interacting domain	Coelho et al., 2014
Protein sequence n-mers (n-gram)	For each pathogen-host protein pair concatenate their vectors. Each protein vector count the number of times each distinct n-mer occurred in the sequence	Dyer et al., 2011
	Similar to Dyer et al. (2011)	Kshirsagar et al., 2012, 2013b
	Variant of the spectrum kernel based on sequence n-mers	Kshirsagar et al., 2013a
	Represent proteins by relative count of amino acid 3-mers	Cui et al., 2012
	Forming 7 amino acid classes and computing frequency difference through 343-dimensional vector	Wuchty, 2011
	Forming 4 amino acid classes and computing standardized frequency difference through 64 possible combination	Dong et al., in press
	Observing each of different 20 amino acids within protein sequence	Coelho et al., 2014
Network topology	Two features for each pathogen-host protein pair including human protein's degree and its betweenness centrality	Dyer et al., 2011
	Three features of human protein: degree, clustering coefficient, centrality	Tastan et al., 2009; Qi et al., 2010; Nouretdinov et al., 2012
	Similar to Tastan et al. (2009)	Kshirsagar et al., 2012, 2013b
	Degree and betweenness centrality in human PPI	Dong et al., in press
Gene ontology	Pairwise similarity between GO terms of host and pathogen and Neighbor similarity for GO terms of pathogen and binding partners of human proteins	Kshirsagar et al., 2012, 2013b
	Pairwise and neighbor GO similarity	Tastan et al., 2009; Qi et al., 2010; Nouretdinov et al., 2012
	Three aspects of Gen Ontology are the only used feature values and the homolog GO features are used for missing data	Mei, 2013, 2014
	Biological process similarity is computed for protein pairs	Coelho et al., 2014
	For every human protein within extracted biclusters find important GO terms	Ray et al., 2012; Mukhopadhyay and Maulik, 2014
	Using GO functional data for conducting two functional analysis	Reid and Berriman, 2013
Gene expression	Differential human gene expression infected by pathogen in seven control conditions	Kshirsagar et al., 2012, 2013b
	Differential human gene expression across HIV-1 infected and uninfected samples	Tastan et al., 2009; Qi et al., 2010; Nouretdinov et al., 2012
Conserved pathways	Find other known PHI, which pathogen is homolog and host proteins share a pathway	Kshirsagar et al., 2012, 2013b
RNAi expression	Utilizing human genes reported as “hits” by the RNAi screens	
Homology information	For each PHI count the number of interologs from other species	
	Forming orthologous groups through clustering host and pathogen proteins around central orthlogous pairs	Wuchty, 2011
	Use STRING to get clusters of orthologous groups and their scores	Coelho et al., 2014
Pfam interactions	Counts the possible interactions between Pfam families of host and pathogen reported in the iPfam	Kshirsagar et al., 2012, 2013b
	Use interacting pair of domains to predict gene interaction between malaria and its hosts (mouse and mosquito)	Reid and Berriman, 2013
Protein sequence	Sequence alignment between pathogen and host proteins computed using PSI-BLAST	Kshirsagar et al., 2012, 2013b
Tissue feature	Check infection susceptibility of tissues	Tastan et al., 2009; Qi et al., 2010; Nouretdinov et al., 2012
Virus protein type	One feature for each HIV-1 protein to compute probability of interacting with human protein	
	A feature vector formed by 11 types of HCV proteins and 9 types of HPV	Cui et al., 2012
Pathways	Pathway participation coefficient is calculated for each protein	Wuchty, 2011
	Use similarity of pathway memberships of human proteins to propose commonality hypothesis across organisms	Kshirsagar et al., 2013b
	For each human protein within extracted biclusters find important KEGG pathways	Ray et al., 2012; Mukhopadhyay and Maulik, 2014
	Find other known PHI, which pathogen is homolog and host proteins share a pathway	Kshirsagar et al., 2012, 2013b

### Handling missing data

Applying machine learning methods and specially supervised learning for situations suffer from data scarcity is challenging. Being limited to well-studied pathogen systems like HIV-1 is the consequence of data dependency. Recently, some solutions are proposed to overcome this limitation by offering substituted values for missing data. For instance, in Kshirsagar et al. (2012) two different methods are proposed including information transfer from other species and model-based imputation. First, they rely on homologous proteins data to provide feature values like GO annotations and gene expression data. This contributes a lot and downgrades the missing data significantly. However, for proteins with no available homolog, they have modeled gene expression value distribution. They have compared the proposed “Cross species imputation” with other imputation techniques. The first method is called “RF” which initiates the missing data to mean value and re-estimate it by choosing the nearest leaf node of the created forest. Another intuitive method is choosing the average of the feature values and the last compared method is discarding any pair with missing value which leads to a reduced dataset. Clear improvements are reported in comparison with the listed imputation methods. It should be noted that using solely statistical methods for estimating features like GO values will be hard due to high dimensionality. Mei (2013) uses homolog information when the features of a protein is unavailable. They have designed various experiments to show the performance of substituting homolog features. Pessimistic experiment, which uses only homolog features to train and test without incorporating any base proteins (called “target” in the article), has promising results, indicating that using homolog information is an effective substitute for the target information to tackle the problem of data unavailability.

### The challenge of non-interacting PPIs

Since there is no available verified non-interacting PPI to be used for training the model, selecting negative data remains as a challenge for PPI prediction. Some studies try to circumvent the obstacle by using methods which do not require negative samples (Ray et al., 2012). However, ignoring non-interacting patterns may increase the rate of false positives (Mei, 2013). The negative set is not defined in Nouretdinov et al. (2012) and instead they use unknown label for other pairs. Most of the studies which formulate the problem as a classification task, have to construct negative class through randomly sampling the data. The rate of positive to negative class is chosen in different manners to avoid biasing classifier toward wrong predictions. A ratio of 1:100 is chosen in Kshirsagar et al. (2012, 2013b) and Tastan et al. (2009) expecting one interaction pair within 100 random pathogen-host pairs. Mei (2013) chooses the same ratio for negative and positive classes, however proposes different idea for choosing negative samples. They put aside sub-cellular co-localized pairs from the negative class and report better performance in comparison with random sampling. The study in Dyer et al. (2011) conducted experiments with different ratios and 10 randomly chosen sets for each ratio and stated that beside clearly different results for different ratios, variability of randomly selected negative samples for each ratio does not have major effect on the result accuracy.

## Homology based approaches

The rationale behind this type of methods is the expectation of conserved interactions between a pair of proteins which have interacting homologs in another species. The conserved interaction is called as “Interolog.” The simple method of identifying Interologs is as follows: Consider a template PPI pair (a, b) in a source species, find the homolog a′ in the host and the homolog b′ in the pathogen, conclude that (a′, b′) interact. Simplicity and clear biological basis are the main advantages of these methods. However, homology to known interactions is not sufficient for evaluating the biological evidence of the predicted results. Different filtering techniques should be considered for assessing the feasibility of the interactions under an *in vivo* condition and consequently decreasing the false positives.

A homology detection method using template PPI databases, DIP (Salwinski et al., 2004) and iPfam (Finn et al., 2014), is published in Krishnadev and Srinivasan (2008) to predict PHI pairs. Searching the sequences of host and pathogen proteins within two template databases are conducted to find a superset of all interactions which are physically and structurally compatible. These potential interactions are refined within two additional filtering steps, to detect biologically feasible interactions including integration of expression and sub-cellular localization data. The authors have applied the same procedure for different pathogens in their subsequent works (Tyagi et al., 2009; Krishnadev and Srinivasan, 2011).

Another research uses the conceptually same approach by exploiting sequence similarity augmented with domain-domain interaction detection (Schleker et al., 2012a). They have two compressive reviews of the computational approaches predicting Salmonella-Host interactions (Schleker et al., 2012b, 2014), which include comparing Salmonella-Human and Salmonella-Plant interaction predictions.

Homolog knowledge can be used indirectly as a remedy for data scarcity and data unavailability by homolog knowledge transfer. Mei (2013) uses homolog information (features) when the features of a protein is unavailable. They have designed different experiments to show the performance of substituting homology features. Pessimistic experiment, which uses only homology features for train and test without incorporating any base proteins (called as “target” in the article) has promising results, indicating that using homolog information is an effective substitute for the target information to tackle the problem of data unavailability.

Another research uses high confidence intra-species PPIs to detect Interologs using ortholog information (Lee et al., 2008). The assumption is that when two orthologous groups are shared between more than two species, there will be a potential Interolog between those orthologous groups. The potential interactions are filtered using gene ontology annotations followed by pathogen sequence filtering based on the presence or absence of translocational signals to refine the predictions. The notable point is negligible intersection of the predicted interactions with those of the reported predictions in Dyer et al. (2007) due to applying different techniques and datasets for same pathogen-host system.

Zhou et al. (2014) introduces the “stringent homology” which does not rely only on intra-species template PPIs to discover interologs and make use of two different organisms as the source of template PPIs to predict PHIs. They also claim that it is not only for the targeted host proteins which tend to be hub in their own PPI network and this is also true about targeting pathogen proteins.

The most important obstacle for using homology based methods is scarcity of available homolog information. For instance, the number of interologs within bacterial PPIs are not dignificant (Kshirsagar et al., 2013b) demonstrating that we cannot rely only on homolog information for every situation without being cautious about data availability. Clearly, it is reasonable to predict more genomic and proteomic data will be available in the future and consequently more accurate homologs are identified paving the way of studying less-known pathogens. Table 3 summarizes the published research for predicting PHIs based on homology information.

**Table 3:** **Homology based approaches for prediction of PHIs**.

**Method**	**References**
Homology detection method using template PPI databases, DIP, and iPfam	Krishnadev and Srinivasan, 2008
Interologs were inferred from ortholog information obtained from high confidence databases	Lee et al., 2008
Homology detection method using template PPI databases, DIP, and iPfam	Tyagi et al., 2009
Homology detection method using template PPI databases, DIP, and iPfam	Krishnadev and Srinivasan, 2011
Introduce stringent homology which uses inter species template PPI	Zhou et al., 2014
Conserved PHI network is generated using interacting proteins of the common conserved inter-species bacterial PPI	Barh et al., 2013
Obtain host-pathogen interactome using sequence and interacting domain similarity to known PPIs	Schleker et al., 2012a
Interolog and Domain based approaches are used to predict PHIs	Li et al., 2012
The ortholog information for the four species are integrated from different databases and interspecies PPI network is constructed followed by dynamic modeling of regulatory responses leads to identifying interactions	Wang et al., 2013

## Structure based approaches

A number of studies are based on structural similarities and use template PPIs to detect similar interacting pairs within host and pathogen proteins. Preliminary ideas presented in Davis et al. (2007) called comparative modeling and was based on their prior work (Davis et al., 2006). Their method starts with a set of host and pathogen proteins and then sequence matching procedures are used to determine the similarities between the host or pathogen proteins with known structure or known interaction protein partners. Sequence similarity score is only used when structure information is unavailable as a statistical potential assessment, to predict interacting partners. Filtering the set of potential interactions is the last step which is performed using the biological contexts of proteins and a network-level filter. The outcome of this process is decreasing the potential PHIs by about five orders of magnitude. The main drawback of this method is that finding high similarity between pathogen proteins and proteins with known structure is not guaranteed for all pathogen proteins. Therefore, unavailability of the spatial structural information would restrict the applicability of this method. Furthermore, they have only the ability to collect limited number of benchmark PPIs from literature to evaluate their prediction performance.

Authors in Franzosa and Xia (2011) claim to significantly reduce the rate of false positives by presenting virus-human structural interaction network, in which, each PPI is associated with a high confidence 3D structural model. Applicability of the method is limited to human-human and virus-human PPIs for which 3D structural models are available. The method starts with extracting human interacting pairs from PDB and followed by mapping virus proteins to them by sequence similarity. They emphasize the importance of constructing a high-resolution, 3D structural view of pathogen-host and within-host PPI networks to discover new principles of PHIs through their review paper in Franzosa et al. (2012).

Another research developed a map of interactions between HIV-1 and human proteins based on protein structural similarity (Doolittle and Gomez, 2010). A comparison of known crystal structures is performed to measure structural similarity between host and pathogen proteins. Human proteins which have high structural similarity to a HIV protein are identified and their known interacting partners are determined as targets. The assumption is that HIV proteins have the same interactions as their human peers. These predicted results refined by two filtering steps using data from the recent RNAi screens and cellular co-localization information. They apply the same method for developing an interaction network between Dengue virus and its hosts (Doolittle and Gomez, 2011). Again, with a similar idea those proteins with comparable structures share interaction partners. The work suffers from the lack of assessment data in a way that, very limited number of used benchmark PPIs are specific to the viral pathogen. Table 4 summarizes the conducted research for predicting PHIs based on structural data.

**Table 4:** **Structure based approaches for prediction of PHIs**.

**Method**	**References**
Comparative modeling of 3D structures	Davis et al., 2007
Sharing interacting partners of structurally similar human proteins to HIV proteins	Doolittle and Gomez, 2010
Structural similarity of Denv proteins to human proteins having known interactions	Doolittle and Gomez, 2011
3D structural interaction network of host-pathogen and within-host PPI networks	Franzosa and Xia, 2011
Assumes that structurally homologous proteins have probably interactors in common	De Chassey et al., 2013

## Domain and motif based approaches

The idea of exploiting domains as building blocks of proteins for predicting PPIs is well-studied for single organisms (Wojcik and Schächter, 2001; Pagel et al., 2004) regarding the fact that domains are the mediators of interactions. The approach presented in Dyer et al. (2007) is one of the pioneer published research for predicting PHIs. However, small list of interactions are presented and their biological relevance are not strongly evaluated. To predict interactions between host and pathogen proteins, they present an algorithm that integrates protein domain profiles with interactions between proteins from the same organism. For every pair of functional domains (d, e) which is present in protein pair (g, h) respectively, the probability of interacting (g, h) is assessed using Bayesian statistics. To apply this idea to a pathogen-host system, they identify domains in every host and pathogen proteins and compute the interaction probability for each pair of host and pathogen proteins that contain at least one domain. Assuming M_g_ as the set of domains contained in protein g the interaction probability of proteins (g, h) is computed as:

$$\text{P}\left(\text{g},\text{h}\right)=1-\prod_{\text{d}\in {\text{M}}_{\text{g}}}\prod_{\text{e}\in {\text{M}}_{\text{h}}}\left(1-\text{P}\left(\text{g},\text{h}|\text{d},\text{ e}\right)\right)$$

The authors have published another study which uses domain profiles as features in supervised machine learning for predicting interactions in HIV-Human system.

A similar knowledge source is chosen in Kim et al. (2007) which makes use of domain information from InterProScan (Quevillon et al., 2005). They predict PPIs using PreDIN (Kim et al., 2002) and PreSPI (Han et al., 2004) algorithms based on domain information. A study for prediction of interacting proteins of rice and *Xanthomonas oryzae* pathovar *oryzae* (Xoo) also uses domain information (Kim et al., 2008). They presented XooNET which provides about 3500 possible interaction pairs as well as the graphical visualizations of the interaction networks.

The work in Arnold et al. (2012) presents a method to predict and rank bacteria-human PPIs based on domain-domain interactions. They collect a list of Pfam domains and bacterial-human proteins which contains one of the listed domains. Then the data was searched for experimentally verified effectors or their homologs in another bacteria. The result is the possible interactions between Salmonella effectors and host proteins.

Not all pathogen systems are appropriate for applying the mentioned domain based approaches, since domains and the related information are not available for all pathogens. For instance, information on domains and the related statistics are not available for a considerable number of the HIV-1 proteins. Regarding this limitation, the work in Evans et al. (2009) concentrates on protein interactions based on short eukaryotic linear motifs (ELMs) for HIV-1 proteins interacting with human protein counter domains (CDs). They do not accept the idea of having relatively weak link among motif/domain bindings and the actual virus-host PPIs which is presented in Tastan et al. (2009). They predict two kinds of interactions for each virus protein, including direct human protein targets (called H1) which bind to virus via a human CD and a virus ELM and the second type includes indirect interactions in which, host proteins that their normal interactions with H1 proteins are potentially disrupted by competition with an HIV-1 protein. Table 5 summarizes the conducted research for predicting PHIs based on domain and motif knowledge.

**Table 5:** **Domain and motif based approaches for prediction of PHIs**.

**Method**	**References**
PreDIN and PreSPI algorithms based on domain information	Kim et al., 2007
Estimating PPI probability using combining interaction probability of domains	Dyer et al., 2007
XooNET uses Structural Interactome MAP (PSIMAP), Protein	Kim et al., 2008
Experimental Interactome MAP (PEIMAP) and Domain-Domain interactions from iPfam	
Based on ELMs on HIV-1 proteins interacting with human protein counter domains (CDs)	Evans et al., 2009
Predict and rank bacteria-human PPIs based on domain-domain interaction	Arnold et al., 2012
Build the virus-host interactomes by identifying domain interactions between virus and host PPIs followed by topological and functional analysis of the network	Zheng et al., 2014
The viral-human interaction network is modeled based on motif-domain interactions	Segura-Cabrera et al., 2013

## Performance evaluation

The lack of gold standard PHI data and the complexity of PHI mechanisms lead to a hard assessment phase, in a way that predicted interactions are rarely supported by a biological basis. Some studies validate their results by measuring the shared interactions with other published materials (Mukhopadhyay et al., 2012, 2014; Segura-Cabrera et al., 2013). Here we focus on computational metrics which are widely used in publications to evaluate the accuracy of their results, which are shown in Table 6.

**Table 6:** **Popular evaluation metrics used for PHI prediction**.

**Metric**	**Formula**	**References**
Accuracy	$\frac{TP+TN}{TP+FP+TN+FN}$	Cui et al., 2012
Specificity	$\frac{TN}{TN+FP}$	Cui et al., 2012
Sensitivity (Recall)	$\frac{TP}{TP+FN}$	Dyer et al., 2011; Cui et al., 2012
Precision	$\frac{TP}{TP+FP}$	Dyer et al., 2011
F1 score	$\frac{2\;*Precision\;*\;Recall}{Precision+Recall}$	Kshirsagar et al., 2012, 2013b; Mei, 2013; Coelho et al., 2014
AUC	The area under the ROC curve	Davis et al., 2007; Mei, 2013; Coelho et al., 2014

*TP, True Positive; TN, True Negative; FP, False Positive; FN, False Negative*.

## Conclusions

Inter-species PPI predictions have gained more popularity in recent years. Computational methods may have important roles in paving the way for experimental PHI verifications by highlighting the high potential interactions and limiting the experimental scope which lead to expense reduction and probably the rapid knowledge development. In this paper, we reviewed the studies which directly focused on computationally PHI prediction. Published approaches are categorized based on pathogen-host and the method they utilize. Clearly some pathogen systems are well studied and targeted in more research regarding the availability of the required data. HIV-1 is the most distinguished pathogen which studied specifically using data-requiring machine learning methods. Therefore, the most important challenge for computationally prediction of PHIs, is the lack of available verified interactions and the relevant feature information in most of the pathogens systems. Data unavailability and scarcity refer to verified interacting PPIs, lack of verified non-interacting protein pairs and missing feature information for proteins. Recent studies have found a new source of data to overcome these limitations. Knowledge transfer from related pathogen systems has shown to be an effective remedy, even for situations with no available interactions. These methods enlighten a promising future direction for establishing computational methods which are augmented with additional transferred knowledge.

### Conflict of interest statement

The authors declare that the research was conducted in the absence of any commercial or financial relationships that could be construed as a potential conflict of interest.
